# Supplementary material for: Learning curve and surgical outcome of robotic assisted colorectal surgery with ERAS program
Source: Sci Rep. 2022 Nov 29;12:20566. doi: 10.1038/s41598-022-24665-w (PMC9709162; doi:10.1038/s41598-022-24665-w)
Supplement: Supplementary file 1 — Supplementary Information 1. [file 41598_2022_24665_MOESM1_ESM.zip › LSC Raw data-Ñ[▒K/051/OP_ñw╝╨░O▒Kñσ.pdf]

正式摘要(電子簽章：000000177778264)

W86 / W86

臺中榮民總醫院

Operation Note

[redacted]

- 手術日期：2019/10/17
- 手術醫師：VS 蔣鋒帆，CR 江世偉，R 林楙時，R 陳映菁
- Anesthesia : GE
- Position : Lithotomy
- Aseptic agents : Betadine Alcohol

Diagnosis

- Pre-operative diagnosis : Malignant tumor of rectum, L/3
- Post-operative diagnosis : Ditto.

Surgical Indication

- Dx and Tx

Operation

- Low anterior resection +/- pelvic LN dissection (side to end anastomosis) Laparoscopy

Finding

- 1. rectal ca, L/3, posterior wall with LAP, tumor size 3x2x2cm, sT3N1M0
  2. smooth liver surface
  3. no obvious IMA root LAP

Procedure

- A Laproscopic anterior resection is done with general anesthesia, with lithotomy position, foley catheter in place. We makes several incision in the lower abdomen to insert the trocar. The position of trocar placement was shoen below. The IMA root was identified and skeleton by harmonic scaple initially. Then IMV was also ligated in the same level. After retroperitoneal dissection , the left side colon and upper rectum was modilized and free from the retroperitoneum, pelvis. The tumor was identified and resected was planned with a safe margin about 10cm in proximal and 5cm in distal. Then we removes the cancer and a margin of normal tissue on either side of the cancer, along with nearby lymph nodes and other tissues around the rectum, by the assisted of endo-GIA 60. the wound around umbilicus was extened to retract the specimen. Then the EEA 29mm was used to reattached to the remaining rectum. After check bleeders mediculously, the penrose drainage tube was placed in the low pelvis. The fascia layer and skin was closed by 1-0 vicryl and 3-0 dermalone. He stood the whole procedure well and sent to POR under stable condition.
- Estimated blood loss: minimum
- 器械數量: 正確
- 紗布數量: 正確

Image

- PAINT
  - colon & rectum

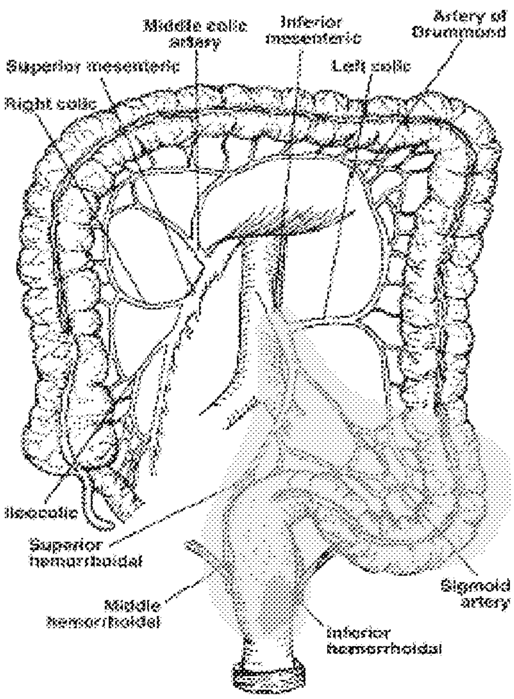

編輯主任 2062F蔣鋒帆

正式摘要(電子簽章：000000177778264)

W86 / W86

doctorRank:B
